# Single-Stage Computed Tomography Angiography, Percutaneous Coronary Intervention, and Transcatheter Aortic Valve Replacement in an Octogenarian With Femoral Neck Fracture, Non-ST-Elevation Myocardial Infarction, and Severe Aortic Stenosis: A Case Report and Literature Review of Multidisciplinary Approaches

**DOI:** 10.31083/RCM50145

**Published:** 2026-09-17

**Authors:** Ming Zhang, Xietian Pan, Lina Yang, Yang Mu, Bo Jiang, Tao Chen, Jing Wang, Lin Wang, Jiayue Li, Jing Liu, Zhuo Zhang, Changfu Liu

**Affiliations:** ^1^Senior Department of Cardiology, The First Medical Center of PLA General Hospital, 100853 Beijing, China; ^2^Senior Department of Cardiology, The Sixth Medical Center of PLA General Hospital, 100853 Beijing, China; ^3^Senior Department of Anesthesiology, The First Medical Center of PLA General Hospital, 100853 Beijing, China; ^4^Senior Department of Orthopedics, The First Medical Center of PLA General Hospital, 100853 Beijing, China

**Keywords:** femoral neck fracture, non-ST-elevated myocardial infarction, aortic valve stenosis

## Abstract

Geriatric femoral neck fractures are associated with high mortality. Early surgery within 48 hours improves survival, whereas delayed arthroplasty is associated with a 41% increase in mortality. Multimorbidity in this population often prolongs preoperative optimization, thereby exacerbating perioperative risk. Current evidence indicates that 5%–10% of patients with hip fractures have concomitant severe aortic stenosis (AS), which independently increases the risk of anesthesia-related complications. Perioperative acute myocardial infarction further increases mortality risk, making cardiac management a critical challenge. The advent of transcatheter aortic valve replacement (TAVR) has revolutionized care paradigms. Nonetheless, this situation requires multidisciplinary consensus on optimizing the fracture-to-surgery interval, antithrombotic bridging strategies, and concomitant coronary revascularization. Here, we present a case of single-stage aorta-coronary computed tomography angiography (CTA), percutaneous coronary intervention (PCI), and TAVR in an octogenarian with femoral neck fracture, severe AS, and non-ST-elevation myocardial infarction (NSTEMI). This pioneering case is accompanied by a systematic review of contemporary management strategies.

## 1. Introduction

Older adults are at increased risk of falls resulting in hip fractures, especially when experiencing cardiac events. Hip fractures represent a major geriatric health burden, with distinct epidemiological patterns. In many cases, they are not just simple fractures. Population-based studies reveal incidence rates of 957 and 414 per 100,000 person-years in women and men aged >65 years, respectively [1]. Globally, hip fractures are projected to reach 4.5 million annually by 2050 (up from 1.26 million in 1990), with 30% of cases arising in China [2,3]. Anatomically, femoral neck fractures constitute 53% of all hip fractures [4]. Current guidelines strongly recommend arthroplasty over internal fixation for unstable (displaced) femoral neck fractures in older adults. Among patients aged over 80 years, cemented hemiarthroplasty is generally preferred, while total hip arthroplasty may be considered in properly selected, higher-demand patients [5]. However, the high prevalence of cardiovascular comorbidities in elderly patients often complicates therapeutic decision-making. Moreover, the substantial cardiovascular risk frequently delays timely surgical intervention for femoral neck fractures [6]. A review of the literature reveals that no published studies have addressed optimal management strategies for femoral neck fractures in patients concurrently presenting with severe aortic stenosis (AS) and non-ST-elevated myocardial infarction (NSTEMI).

## 2. Case Report

An 84-year-old woman was admitted via emergency medical services on April 16, 2022. She presented with acute dizziness and syncope during brisk walking, culminating in a ground-level fall with subsequent left thigh pain preventing ambulation. Emergency assessment revealed tachycardia (101 bpm), low-grade fever (37.6 °C), and hypertension (147/78 mmHg). Cardiopulmonary examination revealed tachypnea with diminished left lung breath sounds, bilateral grade ≥2 basilar crackles, and a Levine 4/6 crescendo-decrescendo murmur at the right upper sternal border. Orthopedic evaluation identified passive positioning with a positive log roll test.

Critical diagnostic findings included electrocardiography showing diffuse ST-segment depression (V3–V6, II) with ST-elevation in aVR, consistent with myocardial ischemia. Radiography confirmed a left femoral neck fracture (Fig. 1). Transthoracic echocardiography revealed severe AS (peak velocity 4.1 m/s, maximum gradient 62 mmHg, mean gradient 36 mmHg) with mild regurgitation, apical regional wall motion abnormalities, moderately elevated pulmonary artery systolic pressure (PASP) (52 mmHg), and preserved left ventricular ejection fraction (LVEF) (50%). Laboratory examinations revealed elevated cardiac troponin T (0.231 ng/mL; reference <0.1 ng/mL), creatine kinase-MB (16.7 ng/mL), and N-terminal pro-B-type natriuretic peptide (NT-proBNP) (3092 pg/mL). Subsequently, the patient was admitted to the Cardiac Intensive Care Unit (CICU) for advanced hemodynamic monitoring and protocolized management.

**Fig. 1.**
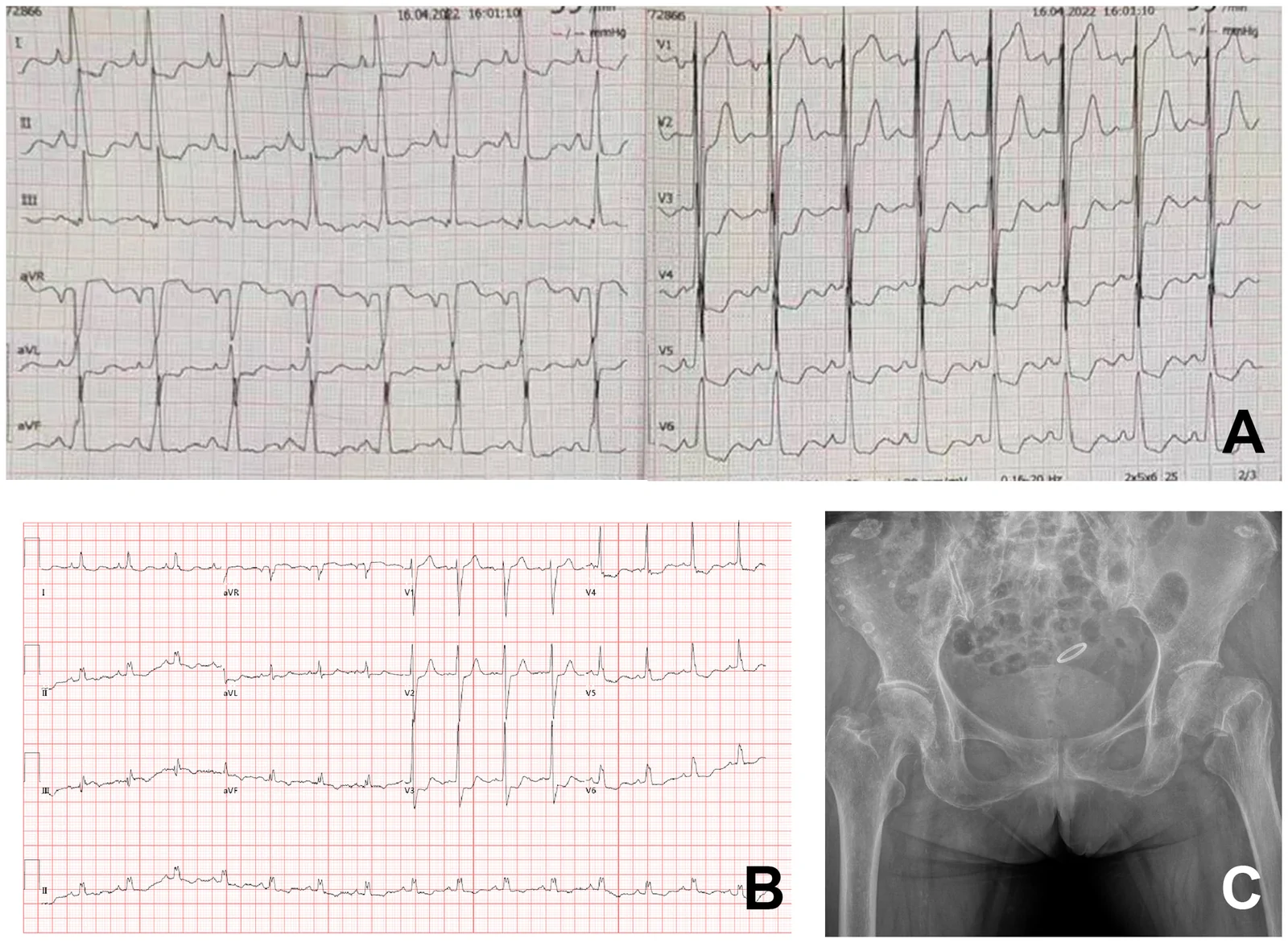
**Representative diagnostic images during hospitalization**. (A) The initial electrocardiogram obtained in the emergency department showed ST-segment depressions in leads V3–V6 and II, with concomitant ST-segment elevation in lead aVR. (B) An electrocardiogram captured during an episode of in-hospital chest pain revealed dynamic ST-segment changes, predominantly in the precordial leads V3–V6. (C) Plain hip radiograph demonstrating a left femoral neck fracture.

The admission diagnosis encompassed: (1) coronary artery disease manifesting as NSTEMI with Killip Class II heart failure; (2) severe AS with mild aortic regurgitation; (3) femoral neck fracture; and (4) stage 3 hypertension with very high cardiovascular risk.

Following admission, the patient maintained passive positioning with persistent severe hip pain despite immobilization, concurrently reporting episodic anginal chest pain characterized by throat constriction. Continuous electrocardiography (ECG) monitoring captured dynamic ST-segment fluctuations in anterior leads (V3–V6) (Fig. 1). Bedside chest radiography revealed pulmonary congestion secondary to cardiac dysfunction, superimposed with emerging infiltrates suggestive of hospital-acquired pneumonia. A multidisciplinary cardio-orthogeriatric team comprising cardiology, orthopedics, anesthesiology, pulmonology, and nephrology specialists implemented a staged management protocol starting with supportive therapy. This included dual antiplatelet therapy, protocolized diuresis, empirical antibiotics, regional neural blockade, and morphine-based analgesia. Subsequently, the patient underwent expedited coronary revascularization and transcatheter aortic valve replacement (TAVR), ultimately paving the way for definitive arthroplasty after hemodynamic stabilization. To minimize pain provocation and cardiovascular risks associated with patient transfer, a single-stage hybrid procedure integrating aortic computed tomography angiography (CTA), percutaneous coronary intervention (PCI), and TAVR was performed on April 27. Immediately post-aortic CTA acquisition, the patient was wheeled through the interconnecting door (<30-second transfer) to the catheterization room, with real-time on-site image analysis proceeding concurrently (Fig. 2). Under general anesthesia with endotracheal intubation, coronary angiography via the right radial artery revealed: (1) diffuse calcified stenosis from the left main to the proximal left anterior descending artery (LAD); (2) 95% stenosis in the mid-LAD segment; (3) 90% ostial stenosis of the left circumflex artery (LCx); and (4) 95% distal stenosis in the right coronary artery (RCA), with chronic total occlusion of the distal LCx. Coronary revascularization was performed based on the angiographic findings: a drug‑eluting stent was implanted in the right coronary artery, while in the left anterior descending artery, which exhibited severe calcification on intravascular ultrasound, only palliative balloon angioplasty was performed, as shown in Fig. 3. Following coronary intervention, transfemoral TAVR was initiated. A temporary pacemaker was deployed via left femoral venous access. A 6-Fr pigtail catheter was positioned at the non-coronary sinus for root angiography via left femoral arterial access. The right femoral artery served as the primary access for valve delivery. After successful wire crossing of the aortic valve, balloon valvuloplasty was performed using a 20 × 40 mm NuMED Z-MED II™ balloon at 4 atm. Subsequently, a 26-mm VenusA-Plus® valve (Venus Medtech) was deployed under rapid pacing (180 bpm) (Fig. 3). Post-procedural transthoracic echocardiography confirmed satisfactory function, with a mean transvalvular gradient of 5 mmHg, and trace paravalvular leak. Post-procedural respiratory failure necessitated prolonged mechanical ventilation. Point-of-care ultrasound (POCUS) revealed bilateral pleural effusions, prompting intensified diuresis (furosemide 10 mg/h IV), broad-spectrum antibiotics (meropenem 1 g Q8H), and therapeutic fiberoptic bronchoscopy for aspiration of tenacious secretions. Despite these measures, extubation failed on day 1. Continuous venovenous hemofiltration was initiated on April 28 to manage fluid overload, achieving net ultrafiltration of 3200 mL over 72 h. By April 30, oxygenation had improved (PaO_2_/FiO_2_ >300 mmHg) with resolved effusions on POCUS, enabling successful extubation. Concurrently, gastrointestinal bleeding manifested on April 29 (positive fecal occult blood, hemoglobin decreased by 2.8 g/L). Antiplatelet therapy was suspended with initiation of pantoprazole infusion (8 mg/h) and enteral rest. After sustained hemostasis (fecal occult blood negative ×3 days), clopidogrel at 75 mg daily was resumed on May 5, followed by low-dose aspirin at 50 mg daily on May 9. A reconvened multidisciplinary consultation (cardiology, orthopedics, anesthesiology, critical care) mandated perioperative antithrombotic bridging. Aspirin/clopidogrel was discontinued on May 16 and substituted with enoxaparin (1 mg/kg SC Q12H). On May 19, definitive orthopedic surgery proceeded via femoral head-neck resection, acetabular preparation, and cemented bipolar hemiarthroplasty (Fig. 4). Total intraoperative blood loss was 200 mL, necessitating transfusion with 4 units of packed red blood cells and 2 units of fresh frozen plasma. Successful extubation was achieved immediately postoperatively. Dual antiplatelet therapy was reinstated on May 22. Postoperative rehabilitation commenced under the supervision of a physiotherapist, and unassisted ambulation with full weight-bearing was achieved at 4-week follow-up. No major adverse cardiac or orthopedic events occurred throughout the 3-month surveillance period.

**Fig. 2.**
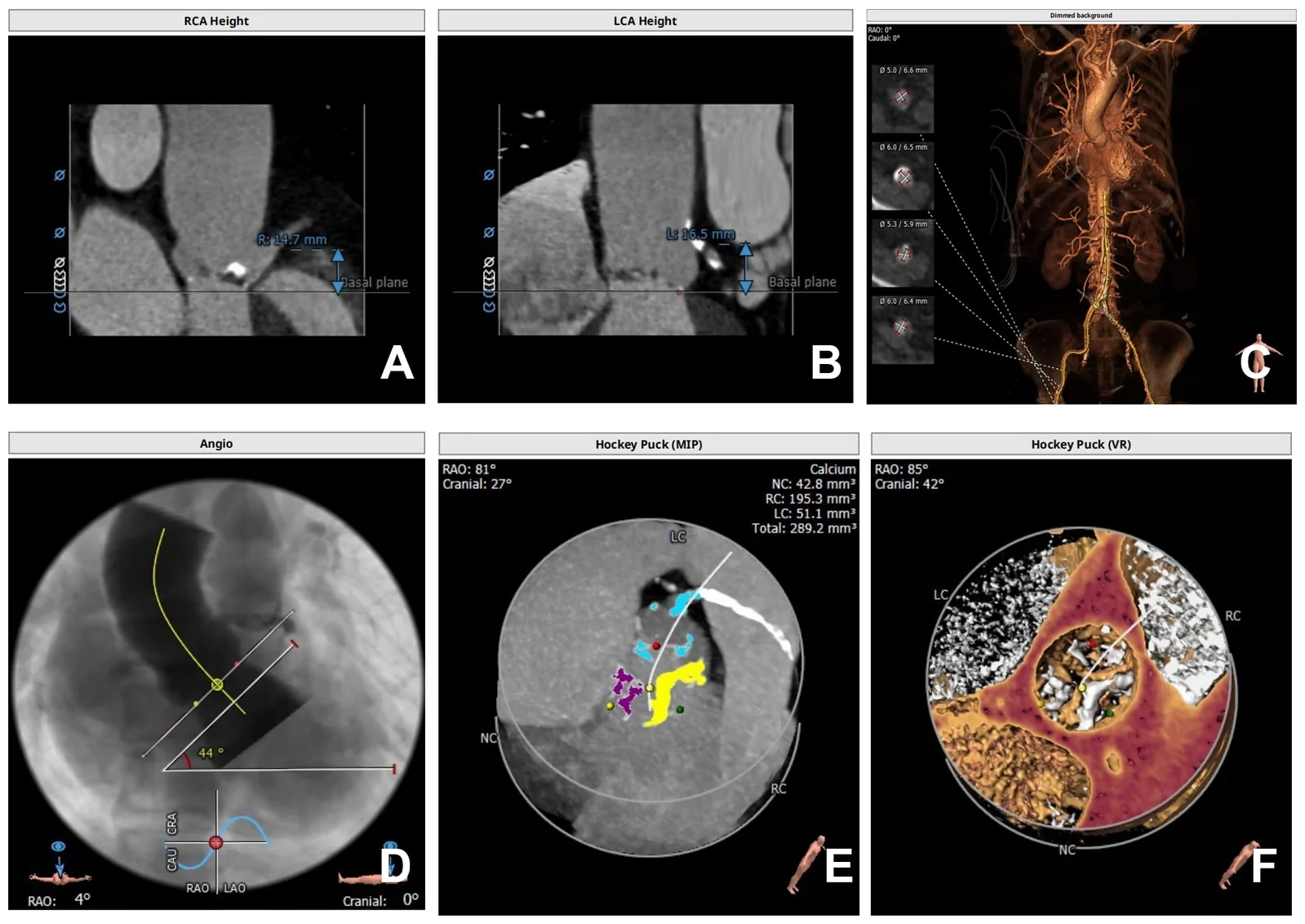
**Pre-TAVR aortic CTA imaging assessment**. (A) The height of the right coronary artery ostium measured 14.7 mm. (B) The height of the left coronary artery ostium measured 16.5 mm. (C) The right femoral artery was selected as the primary access site. (D) The aortic root angulation was 44 degrees. (E,F) The aortic valve shows thickening with associated calcification.

**Fig. 3.**
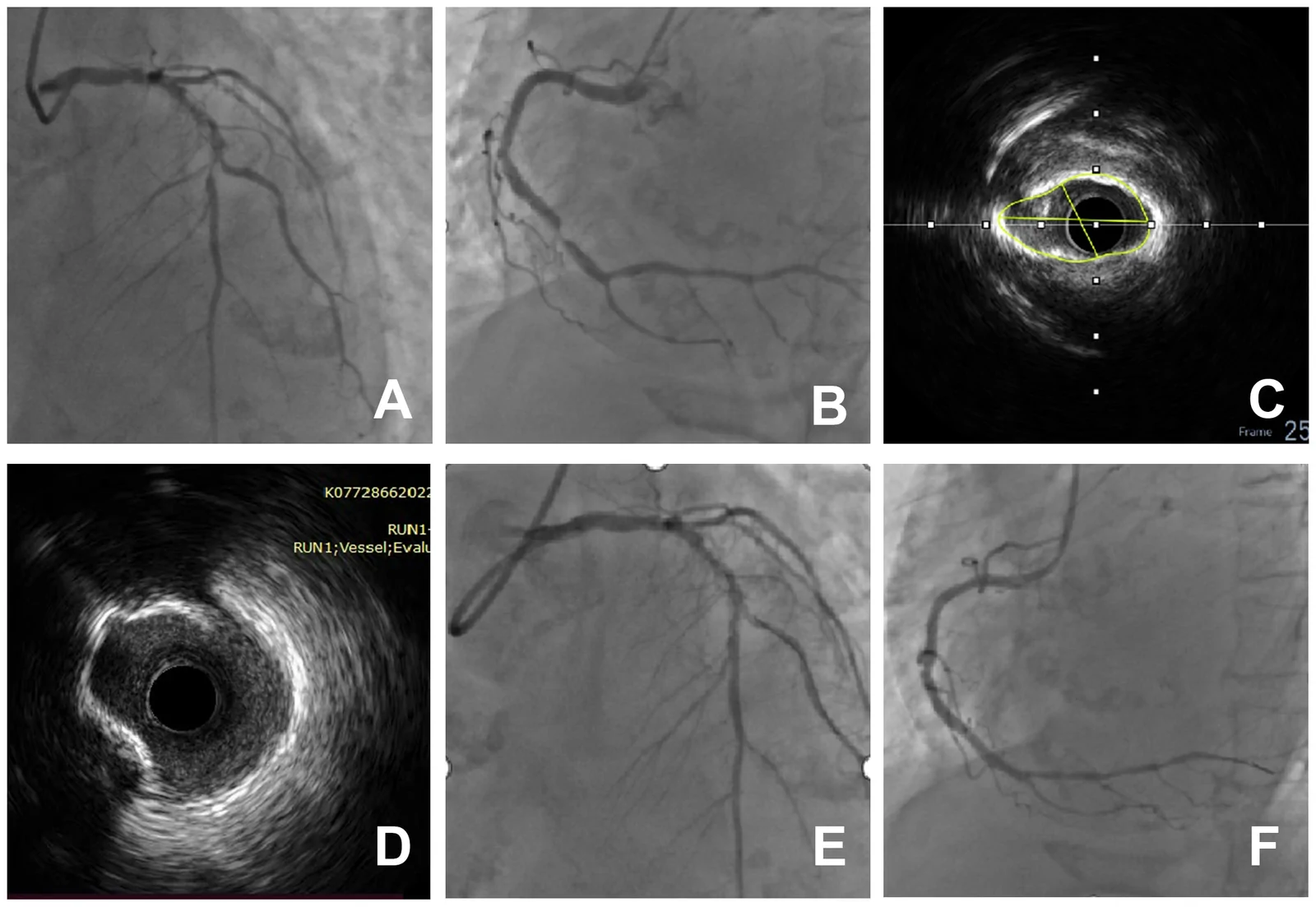
**Coronary angiography findings and intervention**. (A) Left coronary angiography revealed diffuse calcification with severe stenosis extending from the left main coronary artery to the proximal left anterior descending (LAD) artery. (B) Right coronary angiography identified a severe stenosis in the distal segment of the vessel. (C,D) Intravascular ultrasound (IVUS) interrogation revealed a minimal lumen area (MLA) of 3.1 mm^2^ at the LAD ostium and 4.8 mm^2^ in the left main trunk, with predominantly calcified fibrotic plaque from the left main to the LAD. (E) Balloon angioplasty was performed on the LAD using a 2.0 × 15 mm balloon. (F) A 3.5 × 18 mm drug-eluting stent was successfully deployed in the right coronary artery.

**Fig. 4.**
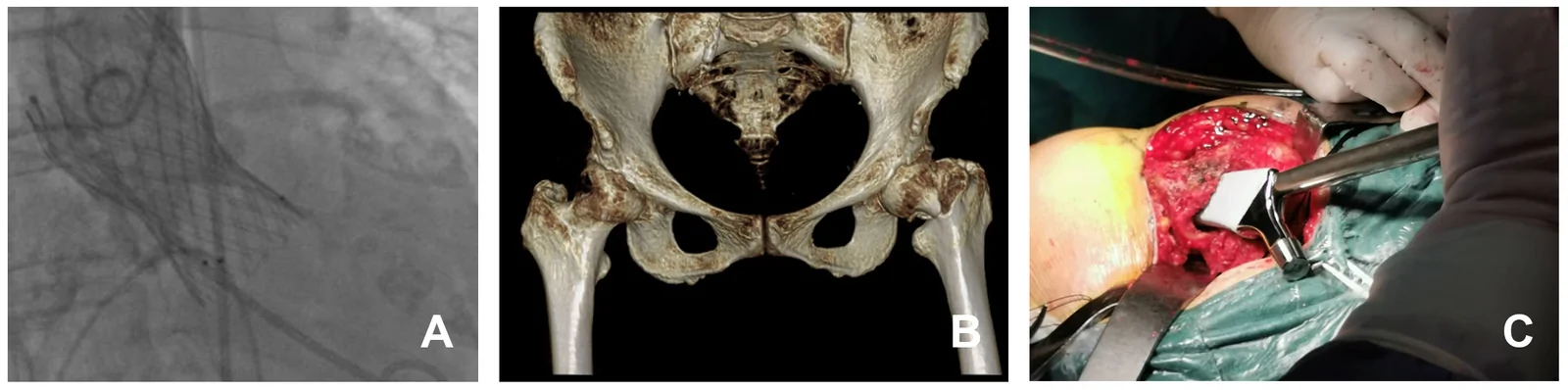
**TAVR and hip arthroplasty**. (A) A 26-mm transcatheter heart valve prosthesis was positioned for deployment during the TAVR procedure. (B,C) A three-dimensional computed tomography (3D-CT) reconstruction depicting the left femoral neck fracture, followed by the implantation of the femoral component during hip hemiarthroplasty.

## 3. Literature Review

To provide a comprehensive overview of the current state of research, we analyzed multiple studies focusing on aortic valve stenosis, NSTEMI and femoral neck fracture. Table 1 (Ref. [7,8,9,10,11,12,13,14,15]) presents a summary of these studies, including their methodologies and key outcomes.

**Table 1.** **Summary of literature review**.

Author	Patient age (yrs)	Number of cases	Clinical conditions	Surgical priority	Anesthetic technique	Result
Keswani et al. [7]	average of 89.2	65 in AS group, 129 in control group	hip fracture & moderate to severe AS	orthopedic procedure	general anesthesia and spinal anesthesia	Moderate to severe AS was an independent predictor of severe perioperative complication, 30-day mortality and 1-year mortality.
Soulioti et al. [8]	96	1	Hip fracture & AS & aspiration pneumonia	Orthopedic procedure	Shamrock lumbar plexus block	Shamrock ultrasound-guided lumbar plexus block combined with sciatic nerve block was a safe and successful anesthetic technique in a frail geriatric trauma patient with comorbidities.
Çağıran et al. [9]	average of 82.2	45	Hip fracture & moderate to severe AS	orthopedic procedure	unilateral spinal anesthesia	Unilateral spinal anesthesia at ultra-low dose is a safe and effective option in elderly patients with moderate to severe aortic stenosis scheduled for hip fracture surgery.
Leibowitz et al. [12]	average of 84.5	32 in AS group, 88 in control group	hip fracture & AS	orthopedic procedure	general anesthesia (10), epidural/spinal (16), peripheral nerve block (3), and N/A (3)	Elderly patients with AS can safely undergo repair of hip fractures, with mortality and morbidity rates comparable with a control population.
Mitsuta et al. [10]	90	1	hip fracture & AS	simultaneous	general anesthesia	TAVI and femoral osteosynthesis were successfully performed simultaneously on a patient with a hip fracture complicated by AS. Careful preparation and perioperative management led to a favorable outcome.
Yamane et al. [11]	90	1	Hip fracture & AS	simultaneous	general anesthesia	Simultaneous and successive surgeries may be an option for patients requiring early recovery and timely rehabilitation
Komarasamy et al. [14]	average of 78.2	11	hip fracture & AMI	orthopedic procedure	spinal anesthesia (5), general anesthesia (4), peripheral nerve block (1), and not recorded (1)	The mortality rate following surgery was 45.4% at 1 month and 63.5% at 6 months.
Thiagarajah et al. [15]	average of 88	25	hip fracture & AMI	orthopedic procedure	N/A	The mortality rate following surgery was 28% at 1 month and 40% at 6 months.
Chaudhry et al. [13]	average of 78	1827	hip fracture & AMI	PCI first vs. orthopedic-first vs. same day	N/A	The in-hospital mortality rate was higher in patients who underwent PCI after orthopedic surgery compared to those who had PCI on the same day or after orthopedic surgery. However, the difference was not statistically significant.

AMI, acute myocardial infarction; N/A, not applicable; PCI, percutaneous coronary intervention; AS, aortic stenosis.

Femoral neck fractures are common conditions in elderly patients, with an annual incidence reaching 6% in those over 80 years old. Women have a 2–3-fold greater risk of femoral neck fractures than men due to their higher prevalence of osteoporosis [16]. Elderly patients with femoral neck fractures experience high mortality rates. Surgical treatment within 48 h of fracture occurrence can reduce mortality, whereas delayed arthroplasty beyond 48 h increases mortality by 41% [17]. However, complex comorbidities in patients with femoral neck fractures can often complicate the treatment process, frequently preventing surgery within the ideal timeframe [18]. Comorbid cardiovascular diseases are present in 35% of hip fracture patients, increasing their mortality risk threefold. Moreover, approximately 2.8–5.1% of femoral neck fracture patients suffer perioperative myocardial infarction [19], and 5–10% have comorbid aortic stenosis [20,21,22]. Moderate to severe aortic stenosis (AS) is an independent risk factor for perioperative complications, 30-day complications, 30-day mortality, and 1-year mortality following hip fracture surgery [18,23]. McBrien et al. [24] reported a 30-day mortality rate of 10% in patients with severe AS who underwent hip fracture surgery, compared to 6.25% in patients with moderate valve disease; despite the substantial difference, it was not statistically significant. Patients with severe AS often have significant coronary artery disease, and approximately 30% of patients undergoing aortic valve replacement were found to have severe coronary stenosis on angiography [25]. In randomized controlled trials, about 12% of TAVR recipients undergo PCI before TAVR, compared to 25% in real-world TAVR registries [26]. Hence, there is significant overlap in the high-risk populations for femoral neck fracture, severe AS, and acute myocardial infarction. Pathophysiologically, these three conditions can promote and influence each other, forming a vicious cycle that becomes a major health threat to the elderly. The catecholamine storm and hypercoagulable state induced by fracture stress can trigger coronary plaque rupture in elderly patients with coronary disease, leading to acute myocardial infarction. severe AS reduces cardiac output and coronary perfusion, significantly increasing the risk of intraoperative hemodynamic collapse during fracture surgery. When all three conditions coexist, multidisciplinary collaboration is required to determine the optimal surgical timing, sequence, anesthesia plan, and perioperative antithrombotic regimen and thus achieve the best possible outcome.

Despite considerable progress in perioperative medicine, patients with severe AS continue to face substantially elevated risks during non-cardiac surgery, with significantly higher rates of major adverse cardiac events [27]. Keswani et al. [7] identified aortic stenosis (AS) as a significant predictor of postoperative complications in elderly patients undergoing hip fracture surgery. In light of this finding, current clinical guidelines advise that elective non-cardiac surgery should be deferred in symptomatic AS patients until after definitive aortic valve intervention [28]. In studies of the optimal timing of orthopedic surgery for patients with comorbid cardiovascular disease and femoral neck fracture, many investigators have focused on optimizing anesthesia techniques to reduce potential cardiovascular risks and ensure the priority and smooth progression of orthopedic procedures. Soulioti et al. [8] reported the case of a 96-year-old hip fracture patient with severe AS and aspiration pneumonia who successfully underwent hip hemiarthroplasty under peripheral nerve blockade as the sole anesthetic technique. Çağıran et al. [9] reported that unilateral spinal anesthesia during hip fracture surgery was safe and effective in geriatric patients with high cardiovascular risk due to AS. A common feature of such cases was that the AS was still asymptomatic, supporting management of the femoral neck fracture as the priority [29]. Some teams opted to perform TAVR and femoral neck fracture surgery simultaneously. Mitsuta et al. [10] reported the case of a 90-year-old woman diagnosed with femoral neck fracture and severe AS. After evaluation by a multidisciplinary team, she successfully underwent simultaneous TAVI and femoral neck fracture surgery under general anesthesia and without significant complications [10]. Similarly, Yamane et al. [11] reported the case of a 90-year-old patient with symptomatic severe AS and femoral neck fracture who underwent TAVR and femoral neck fracture surgery on the same day. Leibowitz et al. [12] reported that elderly patients with severe AS can safely undergo repair of hip fractures, with comparable mortality and morbidity to the control population. With the advent and widespread adoption of TAVR, the role of Balloon Aortic Valvuloplasty (BAV) has diminished. However, for patients with femoral neck fractures who urgently require mitigation of cardiovascular risks, BAV remains a viable option. It serves as a low-risk bridge to stabilize the patient’s hemodynamic status and create a safer window for subsequent orthopedic surgery. While current guidelines and expert consensus have not yet provided specific recommendations on the strategy, Terré et al. [18] summarized that for patients with severe AS and femoral neck fractures, if TAVR is contraindicated, BAV can be used as a bridge. In such cases, definitive TAVR can be performed electively after the completion of the femoral neck fracture surgery.

When formulating a surgical strategy for a femoral neck fracture with comorbid severe AS, the sequence of interventions must be considered very carefully. However, when this is further complicated by frequently recurring NSTEMI, the prioritization of cardiac surgery becomes unquestionable. In a study by Komarasamy et al. [14], patients with hip fracture and acute myocardial infarction undergoing orthopedic surgery had a mortality rate of 45.4% at 1 month and 63.5% at 6 months. Similarly, Thiagarajah et al. [15] reported that a group of patients with hip fracture and acute myocardial infarction who underwent orthopedic procedure had a mortality rate of 28% at 1 month and 40% at 6 months. Chaudhry et al. [13] found that in-hospital mortality for patients with hip fracture and AMI did not differ significantly according to the timing strategy for the PCI, i.e., whether PCI was performed prior to, following, or concurrent with the orthopedic procedure. To the best of our knowledge, cases with the complexity of comorbid hip fracture, severe AS, and NSTEMI have not been previously reported in the literature. The guidelines recommend PCI within 24 h for high-risk NSTEMI patients, and within 2 h for very high-risk NSTEMI patients [30]. Early intervention in high-risk NSTEMI patients can significantly reduce mortality [31]. The concomitant need for antithrombotic therapy due to acute NSTEMI and the inherent bleeding risk of femoral neck fracture surgery demand exceptionally meticulous perioperative antithrombotic management. Guidelines recommend that for patients taking aspirin and clopidogrel, antithrombotic agents should be discontinued for a minimum of 3 days prior to surgery, with low-molecular-weight heparin used as bridge therapy [30]. Antiplatelet therapy should be resumed as soon as possible after the second postoperative day, following a thorough assessment of the bleeding risk.

## 4. Discussion

In the present case, the patient’s fall was triggered by a cardiovascular event. The complex condition was identified upon admission, including femoral neck fracture, severe AS, NSTEMI, and a suspected pulmonary infection, necessitating careful multidisciplinary evaluation and management. During hospitalization, the patient experienced frequent episodes of chest pain accompanied by significant dynamic ST-segment changes on electrocardiogram. Once the patient’s condition was relatively stable, a single-stage CTA, TAVR, and PCI procedure was performed to successfully address the cardiovascular risk factors that precluded non-cardiac surgery. The concomitant CTA, PCI, and TAVR procedure was chosen to provide a “one-stop” solution for this high-risk patient. This approach is supported by current literature as a safe strategy that avoids the risks of a second procedure and ensures revascularization and valve replacement in a single setting [26]. Given the patient’s complex clinical presentation and severe calcification in the LAD, achieving complete revascularization was deemed technically challenging and associated with higher risks. Consequently, balloon angioplasty was performed on the LAD to achieve partial revascularization. Even with the completion of TAVR and PCI, it took one month after admission for the patient to finally undergo orthopedic surgery. The delayed surgery was medically necessary due to the patient’s critical and unstable condition following the initial cardiovascular procedures. The delay was not elective; rather, it was a consequence of managing life-threatening complications. After the PCI and TAVR procedures, the patient experienced a period of hemodynamic instability, followed by episodes of infection and gastrointestinal bleeding. Under these circumstances, performing a major orthopedic surgery (which carries significant blood loss and physiological stress risks) would have posed an unacceptably high risk of mortality or further decompensation. Therefore, the surgical team adopted a conservative, stepwise approach.

This case represents an uncommon and complex clinical scenario in which femoral neck fracture, NSTEMI, and severe AS co-occurred. To date, no clinical guideline provides a clear management pathway for such a triple diagnosis. During the course of treatment, several complications developed, and definitive hip surgery could only be performed one month after the cardiac procedure. Despite these challenges, the patient ultimately achieved a favorable outcome. We do not propose this approach as a new standard of care; rather, we suggest that in carefully selected, complex cases where guidelines are silent, an individualized invasive strategy as described may offer clinical reference value for practitioners facing similar dilemmas.

## 5. Conclusions

For patients with femoral neck fracture complicated by severe AS and acute NSTEMI, the cardiac risk of non-cardiac surgery is extremely high. Therefore, prompt cardiac intervention is necessary to eliminate cardiovascular contraindications. A single-stage CTA, TAVR, and PCI strategy significantly reduces patient transfer-related risks, shortens the preoperative preparation time for subsequent orthopedic surgery, and improves perioperative outcomes.
